# Growth of Medicare Advantage After Plan Payment Reductions

**DOI:** 10.1001/jamahealthforum.2023.1744

**Published:** 2023-06-24

**Authors:** Aaron L. Schwartz, Seyoun Kim, Amol S. Navathe, Atul Gupta

**Affiliations:** 1 Department of Medical Ethics and Health Policy, Perelman School of Medicine at the University of Pennsylvania, Philadelphia; 2 Division of General Internal Medicine, Department of Medicine, Perelman School of Medicine at the University of Pennsylvania, Philadelphia; 3 Center for Health Equity Research and Promotion, US Department of Veterans Affairs, Philadelphia, Pennsylvania; 4 The Wharton School, University of Pennsylvania, Philadelphia; 5 National Bureau of Economic Research, Cambridge, Massachusetts

## Abstract

**Importance:**

Various policy proposals would reduce federal payments to Medicare Advantage (MA) plans. However, it is unclear whether payment reductions would compromise beneficiary access to the MA program.

**Objective:**

To quantify the association between MA payment reductions under the Affordable Care Act (ACA) and MA enrollment growth.

**Design, Setting, and Participants:**

This retrospective cohort study examined the MA market before and after the ACA, which mandated cuts to MA benchmark payment rates. Using 2008 to 2019 county-level enrollment and payment data, a difference-in-differences analysis was conducted comparing MA enrollment changes between counties with larger vs smaller benchmark reductions, before vs after the ACA.

**Main Outcomes and Measures:**

The primary outcome was the MA enrollment rate, defined as the proportion of a county’s Medicare beneficiaries enrolled in MA. A secondary analysis examined MA plan payments per member per month.

**Results:**

Among 3138 counties with 37 639 county-year observations, ACA-induced benchmark cuts were sizeable and varied, ranging from 0% to 42.9% (mean [SD], 5.9% [6.6%]). Counties with benchmark cuts above the 75th percentile had population-weighted average benchmark cuts of 14.9% compared with 4.4% in other counties. In the 8 years following the ACA, there was no differential change in MA enrollment between counties with larger vs smaller benchmark cuts (difference-in-differences estimate, 0.02 [95% CI, −1.18 to 1.21] percentage points; *P* = .98). Plan payments differentially fell in counties with larger benchmark cuts by $78.35 (95% CI, $62.21-$94.48) per member per month (*P* < .001).

**Conclusion and Relevance:**

This cohort study found no evidence that the MA benchmark and ensuing payment cuts imposed by the ACA were associated with reduced MA enrollment, compromising access to MA. This evidence can inform ongoing policy debates regarding the growth of MA, concerns about excess payments to MA plans, and proposed Medicare reforms, including further reductions in MA payments.

## Key Points

**Question:**

Were the Affordable Care Act’s payment cuts to Medicare Advantage plans associated with differential reductions in Medicare Advantage enrollment?

**Findings:**

In this cohort study using a difference-in-differences analysis of 3138 counties with 37 639 county-year observations, during the 8 years following the Affordable Care Act, counties with larger cuts to Medicare Advantage plan payments had similar Medicare Advantage enrollment growth as counties facing smaller cuts.

**Meaning:**

Payment cuts of the magnitude imposed by the Affordable Care Act did not appear to reduce enrollment growth in Medicare Advantage; modest plan payment cuts may reduce federal spending without compromising access to Medicare Advantage.

## Teaser

This cohort study examines the association between the Affordable Care Act’s Medicare Advantage payment reductions and Medicare Advantage enrollment growth.

## Introduction

Medicare Advantage (MA) is forecast to encompass half of overall Medicare enrollment by 2023, a new milestone in the vast expansion of private health insurance within Medicare.^1^ This growth has fueled concerns that excessive federal payments to MA plans may be driving this trend. The MA risk-adjustment system, which scales MA plan payments based on enrollee comorbidities, has attracted particular criticism,^2,3^ prompting new expansions to federal oversight via audits.^4^ The Medicare Payment Advisory Commission estimates that payments to MA plans are several percentage points higher than the expected spending of MA beneficiaries had these beneficiaries enrolled in fee-for-service (FFS) Medicare.^5^ Greater payments to MA plans may allow MA plans to boost enrollment by offering more extensive benefit offerings than FFS Medicare. This association between the generosity of plan payments and MA enrollment has been documented in several studies that examined idiosyncratic variation in payment generosity.^6,7,8,9,10^

However, curbing MA plan payment could bring unintended consequences. The generosity of MA plan payments can finance supplemental benefits, such as premium reductions for prescription drug coverage, dental coverage, and gym memberships, and may promote health care quality. In quasi-experimental and descriptive studies, MA has generally performed as well or better than FFS Medicare on some measures of health care quality.^11,12^ For example, MA appears to reduce hospitalization rates,^6,13,14,15^ without evidence of worsened mortality.^6,16^ Heart attack mortality had historically been lower in MA, though these mortality rates equalized over time.^17^ In addition, MA has also tended to perform well with respect to outpatient process measures of quality.^11^ Thus, it is possible that policy reforms to MA plan payment could reduce beneficiary access to valuable benefits.

To inform this important debate, we studied a policy change in the Affordable Care Act (ACA) that reduced MA payments in different US counties to differing degrees. The ACA reforms to MA payment, arguably the most substantial MA reforms since the Medicare Modernization Act of 2003,^5^ served an important role as a source of financing for the ACA; in evaluating the budgetary influence of the legislation, the Congressional Budget Office projected that ACA reforms to MA would reduce federal spending by $136 billion from 2010 to 2019.^18^ The main policy mechanism for reducing MA plan payments was a revision to the methodology for calculating county benchmarks, a key element determining plan payment levels. Although these reforms did not appear to shrink the MA market immediately,^19^ little is known about their long-term consequences. Understanding the implications of payment cuts for MA growth could inform policy makers about the trade-off between promoting access to MA and containing governmental spending. In this retrospective cohort study, we examined the association between MA plan payment cuts from the ACA and MA enrollment.

## Methods

### Data Sources and Sample Population

This analysis used 2008 to 2019 Centers for Medicare & Medicaid Services’ public files, including MA ratebooks, MA state/county penetration files, and Part C plan payment files, which collectively contain county-level enrollment, benchmark, and payment data.^20,21,22^ We did not extend the analysis beyond 2019 to avoid data derived during the COVID-19 pandemic. We excluded a single county-year that reported greater than 100% MA enrollment. The research protocol was exempt from institutional review board review because it used only publicly available, aggregate data.

### Quantifying Benchmark Changes

Broadly, during the study period, 3 key factors determined the payments received by MA plans: (1) a county-level benchmark, (2) a plan-level bid, and (3) the characteristics of enrolled MA beneficiaries. Benchmarks reflect the maximum amount the government will pay an MA plan monthly for a typical beneficiary. Benchmarks are set based on FFS Medicare spending; the ACA modified the formula determining these benchmarks. Bids, which are submitted by plans, serve as the insurers’ price quotes for providing standard Part A and Part B benefits to a typical beneficiary. Bid levels and their difference from county benchmarks determine not only the plan payment level, but also whether enrollees must face an MA enrollment premium and whether the plan receives an additional rebate payment that can be used to finance additional MA benefits or reduce beneficiary Medicare premiums (ie, for Part D, Part B, or supplemental benefits in their MA plan). Finally, plan payments are risk adjusted to account for beneficiary characteristics that are associated with higher or lower medical spending.

The ACA’s new MA payment formula included benchmark cuts targeted at counties with greater historical Medicare spending. The post-ACA benchmark formula introduced new adjustment factors based on a county’s quartile of per-capita FFS spending; counties with higher spending received benchmarks that were a lower fraction of spending.^23^ Furthermore, a county’s post-ACA benchmark level was capped such that it could be no greater than a benchmark that would be calculated using the pre-ACA methodology. Consider the case of 2 LaSalle counties. In 2011, LaSalle, Illinois, and LaSalle, Texas, had respective benchmark levels of $737 and $1186 per beneficiary per month; in 2012, the respective post-ACA benchmarks for these counties were $759 and $730 per beneficiary per month. However, these benchmark changes were phased in over several years, during which time the effective benchmarks in each county were a blended mix of the pre-ACA and post-ACA benchmark calculations. All counties transitioned fully to ACA-set benchmarks by 2017. The reforms coincided with a reduction in average plan payments from approximately 112% of FFS spending in 2011 to 103% of FFS spending in 2019.^5^ See the eMethods in Supplement 1 for additional details of the reforms.

To quantify the magnitude of payment reductions, we calculated each county’s ACA-induced benchmark cut. We calculated this quantity as the difference between a county’s 2017 benchmark using the pre-ACA and post-ACA formula as a proportion of the pre-ACA 2017 benchmark. We examined 2017 benchmark changes because this was the first year that ACA benchmark calculations were fully in effect nationwide. Because 2017 benchmarks from the pre-ACA formula were included in the data source, we did not need to simulate counterfactual benchmark calculations. Because county benchmark changes depended on the county’s quartile of FFS spending, we grouped counties in the highest quartile of benchmark cuts in the primary analysis.

### Outcomes

The primary outcome, the county-level MA enrollment rate, was calculated yearly as MA enrollment divided by total Medicare enrollment for the month of June, which avoids the MA open enrollment period spanning January to March. For 960 county-year observations whose enrollment data were masked due to fewer than 10 MA enrollees, we imputed an MA enrollment rate of 0%.

The secondary outcome was federal MA plan payment levels. The measure of plan payment includes not only base payment rates, but also risk adjustment for beneficiary characteristics and additional rebate payments. To focus the analysis on government payment generosity, we did not include beneficiary premiums in this measure. Because benchmark levels are one of several parameters determining plan payment, the ACA benchmark reforms did not guarantee that MA plan payments would fall in targeted counties. Payment levels might have been stabilized by counteracting actions of insurers, such as changes to bidding behavior, or by other features of the ACA reforms, such as a new quality bonus program.^23^ To visualize payment changes over time, we generated a weighted average of plan payments per member per month, weighted by estimates of plan enrollee count. See the eMethods in Supplement 1 for additional details.

### Statistical Analysis

We conducted a difference-in-differences analysis to compare changes in MA enrollment and plan payments following the ACA for counties experiencing larger benchmark cuts (75th percentile or above) vs smaller benchmark cuts (all other counties). We modeled the phase-in period of 2012 to 2016 as a washout period, assessing the post-ACA enrollment outcomes beginning in 2017 after the phase in of post-ACA benchmarks. Robust standard errors were clustered at the county level. We weighted county-year observations by Medicare population using the total number of county Medicare enrollees in 2011. In sensitivity analyses, we used alternate definitions of larger vs smaller benchmark cuts, used continuous measures of benchmark cuts, adjusted for any pre-ACA nonparallel temporal trends in outcome, considered the washout period as part of the post-ACA period, and conducted unweighted analyses.

In the difference-in-differences analysis of the secondary outcome, plan payment levels, the unit of analysis was the county-year-plan type (ie, with 1 observation for 2013 preferred provider organization plans in LaSalle County, Texas). See the eMethods in Supplement 1 for additional details.

All analyses were conducted in Stata, version 16.1 (StataCorp). All statistical tests were 2-sided, with *P* < .05 considered statistically significant.

## Results

**Figure 1.**
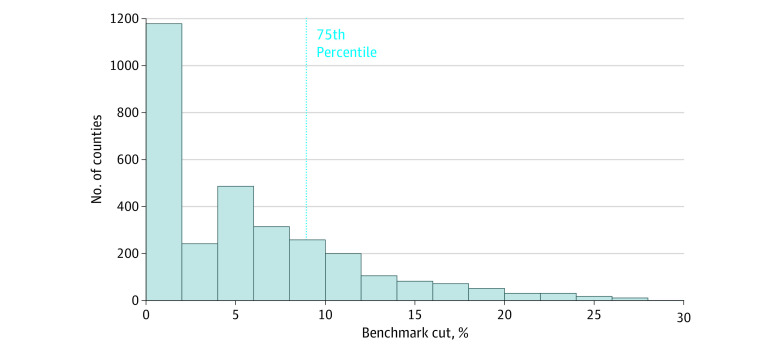
Distribution of Affordable Care Act–Induced Cuts to Medicare Advantage Across US Counties In 2017, the mean benchmark was $827.37. The mean benchmark cut was 5.9%. The highest 1% of Affordable Care Act–induced benchmark cuts was excluded to improve readability of the Figure.

The sample included 3138 counties and 37 639 county-year observations with a mean (SD) 2017 benchmark of $827.37 ($53.32) per beneficiary per month. The ACA-induced benchmark cuts were sizeable and varied, ranging from 0% to 42.9% (mean [SD], 5.9% [6.6%]; population-weighted mean [SD], 9.0% [6.8%]; Figure 1). Counties with benchmark cuts above the 75th percentile (8.9%) had population-weighted mean benchmark cuts of 14.9% (unweighted mean, 14.9%), while other counties had mean cuts of 4.4% (unweighted mean, 2.8%). Counties with larger cuts had greater MA enrollment rates at baseline than other counties (Table 1).

**Table 1.** County Characteristics^a^

Characteristic	Mean (SD)		
	All counties (n = 3144)	Counties with largest cuts (n = 786)	Counties with smaller cuts (n = 2358)
Benchmark in 2017, $			
Post-ACA	823.29 (42.36)	821.13 (49.35)	824.96 (35.92)
Pre-ACA	910.66 (95.75)	970.54 (106.37)	864.18 (50.58)
MA enrollee count, No.			
In 2011	40 506 (79 239)	67 920 (107 356)	19 226 (34 205)
In 2019	70 497 (130 322)	113 382 (175 475)	37 177 (60 965)
MA enrollment rate			
In 2011	0.25 (0.14)	0.30 (0.14)	0.21 (0.13)
In 2019	0.37 (0.14)	0.42 (0.13)	0.33 (0.13)
MA per member per mo payment, $			
In 2011	801.38 (132.84)	870.19 (134.23)	747.94 (104.06)
In 2019	950.61 (100.26)	973.71 (99.09)	932.62 (97.44)
Region, %			
Midwest	22.6	9.5	32.9
Northeast	19.3	24.4	15.3
South	37.4	39.0	36.2
West	20.7	27.2	15.6

Abbreviations: ACA, Affordable Care Act; MA, Medicare Advantage.

^a^ Observations are weighted by 2011 county Medicare enrollment.

**Figure 2.**
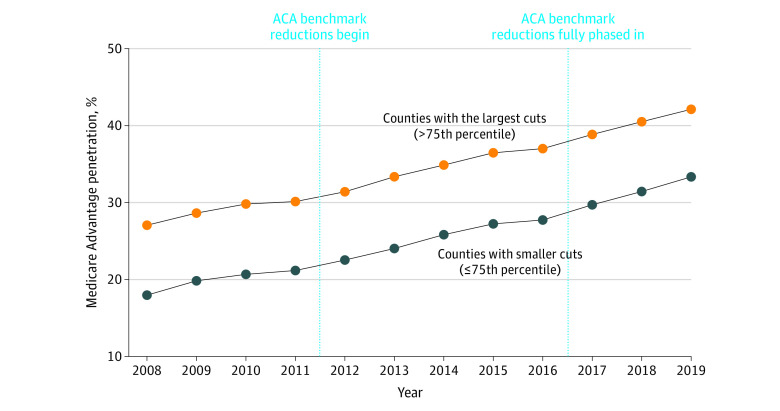
Trends in Medicare Advantage Enrollment by Size of Medicare Advantage Benchmark Cuts Medicare Advantage enrollment is the share of Medicare beneficiaries in a county who are enrolled in Medicare Advantage. Average Medicare Advantage enrollment rate is shown between 2008 and 2019 by the magnitude of county benchmark cuts. Observations are weighted by 2011 county Medicare enrollment. Counties are categorized according to whether they experienced Affordable Care Act (ACA)-induced benchmark cuts greater than or equal to the 75th percentile.

Larger benchmark cuts were not associated with lesser growth in MA enrollment (Figure 2 and eFigure in Supplement 1). The primary difference-in-differences estimate indicated that, following the ACA, there was a statistically insignificant 0.02 (95% CI, −1.18 to 1.21) percentage point greater increase in MA enrollment for counties with larger vs smaller cuts (*P* = .98). Sensitivity analyses, including models adjusting for any differential pre-ACA enrollment trends, also failed to detect a negative association between benchmark cuts and enrollment growth (Table 2 and eTable 1 in Supplement 1).

**Table 2.** Difference-in-Differences Estimates of the Associations Between ACA Reforms and MA Enrollment and Payment

Counties in higher benchmark cut group	Higher benchmark cut counties		Lower benchmark cut counties		Unadjusted difference-in-differences	Regression estimates of difference-in-differences	
	Pre-ACA	After phase in	Pre-ACA	After phase in		Coefficient (95% CI)	*P* value
**Outcome: MA enrollment, %**							
Top 25%	28.9	40.6	19.9	31.5	0.019	0.017 (−1.175 to 1.210)	.98
Top 50%	26.2	37.9	16.7	28.1	0.344	0.341 (−0.607 to 1.290)	.48
Top 10%	30.7	42.5	22.4	34.0	0.226	0.231 (−1.596 to 2.057)	.80
**Outcome: MA payment, $ per beneficiary per mo**							
Top 25%	873.55	933.56	761.13	896.68	−75.535	−78.348 (−94.482 to −62.213)	<.001
Top 50%	843.16	929.93	708.92	851.52	−55.829	−57.102 (−71.220 to −42.984)	<.001
Top 10%	958.82	951.80	783.02	906.13	−130.134	−132.732 (−159.079 to −106.385)	<.001

Abbreviations: ACA, Affordable Care Act; MA, Medicare Advantage.

**Figure 3.**
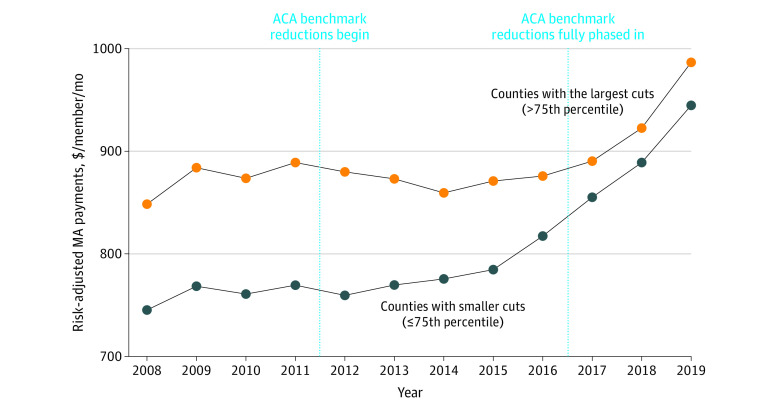
Trends in Medicare Advantage (MA) Payment by Size of MA Benchmark Cuts Risk-adjusted MA payment (amount per member per month) is the enrollment-weighted average federal payment to MA plans each year, shown by the magnitude of county benchmark cuts. County plan type observations are weighted by estimated MA enrollment. Counties are categorized according to whether they experienced Affordable Care Act (ACA)-induced benchmark cuts greater than or equal to the 75th percentile.

The phase in of ACA reforms corresponded to a substantial relative reduction in MA plan payments for counties with larger benchmark reductions (Figure 3). From 2008 to 2011, counties with benchmark cuts above the 75th percentile had enrollment-weighted, risk-adjusted average payments that were $112.42 per member per month higher than other counties. From 2017 to 2019, after the ACA benchmark changes were fully phased in, the difference narrowed to $36.88 per member per month (Table 2). The primary difference-in-differences estimate indicated that the phase in of benchmark reductions was associated with a $78.35 (95% CI, $62.21-$94.48) differential reduction in risk-adjusted MA plan payments for counties with larger vs smaller cuts (*P* < .001). Again, sensitivity analyses yielded similar results (Table 2 and eTable 2 in Supplement 1).

## Discussion

This study did not find evidence of reductions in MA enrollment associated with the MA plan payment reforms of the ACA. In the 8 years after the policy change, this analysis confirmed that the counties most affected by the reforms, as measured by benchmark reductions, experienced relative reductions in plan payment levels compared with counties facing smaller benchmark cuts; however, they experienced similar MA enrollment growth. This study extends prior research showing no immediate effects of the ACA on MA enrollment in the 1 year after ACA reforms.^19^ The present results suggest that MA enrollment was not sensitive to payment cuts of the magnitude imposed by the ACA (ie, roughly 15% benchmark cuts for counties in the top quartile, roughly 11 percentage points greater than other counties) and that recent MA growth likely reflects a multitude of secular trends.

This study can inform ongoing policy debates regarding the growth of MA, concerns about excess payments to MA plans, and proposed Medicare reforms including further changes to MA benchmarks.^1,2,3,4^ One interpretation of these findings is that the MA program could absorb further payment cuts without shrinking the MA market. Indeed, the present results provide direct evidence that ACA-induced payment reductions did not curb MA enrollment. This finding also provides indirect evidence that the ACA payment reductions did not compromise benefits highly valued by Medicare beneficiaries who were deciding whether to enroll in MA. However, we cannot rule out the possibility that the ACA’s payment cuts reduced the value of MA plan offerings but that these changes did not affect overall MA enrollment. For example, MA insurers might have reduced the generosity of certain benefits that prospective enrollees undervalue (perhaps inappropriately) or that are valued only by inframarginal MA enrollees (ie, those who would enroll in MA even if MA were considerably less desirable). Under these circumstances, Medicare beneficiaries could be worse off even without changes in MA enrollment. These possibilities should be investigated in further research examining changes in MA benefit offerings after the ACA. Finally, it is important to note that the enrollment effects of future MA payment reductions, even of the same magnitude, could differ from those observed following the ACA; if MA plan payments fell to a greater degree, then more striking reductions in benefit generosity and enrollment might occur. Also, payment cuts may have different effects depending on which aspect of the payment calculations is modified (eg, benchmark rate vs risk-adjustment calculations).

This study’s key findings differ from prior research showing associations of idiosyncratic variation in MA benchmark levels with changes in MA enrollment.^6,7,8,9,10^ For example, several studies have examined the association between increased MA payments for urban counties and increased MA enrollment.^6,7,8,9^ These studies focused on quasi-experimental variation in plan payments arising from a 2003 policy regarding so-called urban floor counties; counties received higher MA plan payments if they were part of metropolitan statistical areas with populations of 250 000 or more and had relatively low FFS spending. Other research has demonstrated an association between MA plan payments and MA enrollment by examining variation in benchmark payments that were uncorrelated with county characteristics and local FFS spending.^10^

The divergence between the present findings and past research may reflect distinctions between the ACA reforms and previously studied sources of MA plan payment variation. For example, we studied payment reductions while others studied payment increases due to the urban floor feature of plan payment calculations. It is possible that insurers respond asymmetrically to payment cuts and payment increases, passing on the benefits of payment increases to consumers to attract enrollment but shielding existing benefits from the payment cuts. Insurers’ response to the ACA may have been muted by the timing of the reform, which occurred after many years of rapid national MA growth and featured a multiyear phase in of new benchmark rates. The ACA also targeted payment cuts to counties with higher benchmark levels and enrollment at baseline. Insurers in these markets may have been uniquely able to absorb payment cuts without compromising benefit generosity (eg, because of strong consumer demand or entrenched insurer investment). These mechanisms could be elucidated in future research quantifying whether some insurers responded to ACA payment reforms via changes in benefit offerings, bid levels, or other behaviors, such as increased coding of beneficiary diagnoses, and whether any of these insurer responses prompted changes in beneficiary demand for MA plans.

### Limitations

This study has several notable limitations. First, the difference-in-differences analysis required a central assumption that, in the absence of the ACA-induced payment cuts, MA enrollment would have changed over time similarly between the counties with larger benchmark reductions and other counties. We did not detect differential MA enrollment trends in the pre-ACA period, which suggests that enrollment trajectories were similar. Second, the results would be subject to bias if the ACA coincided with simultaneous changes in other factors that influenced MA enrollment differently in treatment and control counties. The ACA entailed a multitude of policy changes; however, we are not aware of other contemporaneous policy changes that would have affected MA enrollment differentially in counties facing payment cuts. Third, as discussed previously, it remains unclear why benchmark reductions were not associated with slowed MA enrollment growth. However, we confirmed that benchmark cuts were associated with substantial relative reductions in MA plan payment amounts. This suggests that the ACA benchmark reductions indeed reduced plan payments in a manner that was not reversed by MA plan responses to the ACA or other aspects of the ACA’s plan payment reforms.

## Conclusions

This retrospective cohort study found that the ACA’s MA payment reforms were not associated with reductions in MA enrollment. Counties that faced greater ACA-induced benchmark reductions, and therefore greater cuts to MA plan payments, experienced similar MA enrollment growth as other counties. In the context of the ongoing debate regarding the size and federal costs of the MA program, these findings suggest that prior reforms were associated with lower federal spending on MA without notably compromising beneficiary access to the MA market. Further research could illustrate what features of the MA marketplace prevented these payment cuts from curbing enrollment growth and investigate if consumer welfare was affected in other ways.

## Notes

Supplement 1.
eMethods

eReferences
**eTable 1.** Enrollment Sensitivity Analysis **eTable 2.** Payment Sensitivity Analysis **eFigure.** County MA Growth Versus Benchmark Cuts

Supplement 2. Data Sharing Statement
