# Supplementary material for: Growth of Medicare Advantage After Plan Payment Reductions
Source: JAMA Health Forum. 2023 Jun 24;4(6):e231744. doi: 10.1001/jamahealthforum.2023.1744 (PMC10290750; doi:10.1001/jamahealthforum.2023.1744)
Supplement: Supplement 1. — eMethods eReferences eTable 1. Enrollment Sensitivity Analysis eTable 2. Payment Sensitivity Analysis eFigure. County MA Growth Versus Benchmark Cuts [file jamahealthforum-e231744-s001.pdf]

Supplemental Online Content

Schwartz AL, Kim S, Navathe AS, Gupta A. Growth of Medicare Advantage after plan payment reductions. *JAMA Health Forum*. 2023;4(6):e231744. doi:10.1001/jamahealthforum.2023.1744

eMethods

eReferences

eTable 1. Enrollment Sensitivity Analysis

eTable 2. Payment Sensitivity Analysis

eFigure. County MA Growth Versus Benchmark Cuts

This supplemental material has been provided by the authors to give readers additional information about their work.

© 2023 Schwartz AL et al. *JAMA Health Forum.*

eMethods

Benchmark Calculations

Our calculation of ACA-associated benchmark changes reflected the timing and mechanism of the ACA reforms. Before the ACA, county benchmarks were set as the greater of either the previous year's benchmark multiplied by the growth in national overall Medicare spending or the projected per capita county FFS spending. The ACA reset county benchmarks at a percentage of the projected per capita county FFS spending that was used before the ACA. The percentage factors were determined by quartile of projected per capita county FFS spending and were set at 95%, 100%, 107.5% and 115%, with the lowest quartile spending counties received the largest percentage factors. To ease the financial transition for MA insurers, a transition period smoothed the payment changes over several years. Depending on the difference between the ACA and pre-ACA rates, each county was given a two, four, or six-year transition period where the effective benchmarks would be a blended mix of the pre-ACA and post-ACA benchmarks. Counties with larger differences were given longer transition periods. All counties transitioned fully to ACA-set benchmarks by 2017.

For each county, we calculated a single quantity representing the magnitude of the ACA-associated benchmark cuts. We refer to this quantity as the ACA-induced benchmark cut. This quantity was calculated for each county as $\frac{B_{2017}^{pre}-B_{2017}^{post}}{B_{2017}^{pre}}$ where B_{2017}^{post} is the 2017 benchmark calculated using the new ACA methods and B_{2017}^{pre} is the counterfactual 2017 benchmark calculated using the pre-ACA methods. We did not simulate the counterfactual benchmark for each county because CMS includes this counterfactual benchmark value in its public data.

Sample Construction and Weighting for Secondary Payment Analysis

The sample construction necessarily differed between our primary enrollment analysis and our secondary payment analysis for two reasons. First, CMS Part C Plan Payment files are organized at the year-county-plan type level. Second, while enrollment and benchmark data are complete for counties with zero MA plan participation, payment data are not complete for such counties.

Thus, our sample included year-county-plan type combinations present in the 2008-2019 Part C Plan Payment files, excluding those with missing payment rates. Furthermore, because we used 2011 enrollment data to generate weights for county-plan type observations, we excluded counties with zero MA enrollment in 2011. This yielded 134,821 year-county-plan type observations, representing 35,763 county-years.

Because MA enrollment can vary widely across county and plan type, we weighted county-plan type observations by an imputed estimate of MA enrollment that was based on 2011 MA enrollment data. Briefly, we constructed weights based on the county-specific count of MA enrollees and the national proportions of MA enrollees in each plan type. For counties that have enrollment in every MA plan type at some point during the panel, the weight for each county-plan type was simply the product of the total county MA enrollment and plan type's proportion

© 2023 Schwartz AL et al. *JAMA Health Forum.*

of national enrollment. If a plan type was not present (i.e. not offered or no enrollees) in a county's panel, the plan-specific enrollment proportions were normalized to sum to 100% across plan types offered in the county's panel.

To calculate national enrollment proportions for each plan-type present in our payment data, we used three data sources: the CMS Monthly Contract and Enrollment Summary Report, the CMS Monthly Enrollment by Contract and the CMS Special Needs Plan Comprehensive Report[1,2,3]. Plan type categories were not consistent between payment and enrollment sources (see crosswalk below). Thus, different data sources were used to calculate national enrollment counts for different plan types in the payment data. We used the CMS Monthly Contract and Enrollment Summary to determine the national enrollment in MSA, PFFS, and Regional PPO plans. We used the CMS Monthly Enrollment by Contract to determine the national enrollment across all HMO and local PPO plan types. And finally, we used the SNP Comprehensive Report to determine enrollment in the various HMO, local PPO, and Regional PPO SNP plans. The HMO and local PPO plan enrollment was calculated as the difference between enrollment in all HMO and local PPO plans and their respective SNP plans. The national enrollment counts for these 13 plan types was used to calculate national proportional ratios, which were then applied for each county, normalized based on which plan types were offered in the county's panel.

Plan Type Crosswalk

Payment Data Plan Type	Monthly Summary Report Plan Type	Monthly Report by Contract Plan Type	SNP Enrollment Plan Type
HMO	-	HMO	-
HMO Chronic or Disabling Condition	-	HMO	HMO Chronic or Disabling Condition
HMO Dual-Eligible	-	HMO	HMO Dual-Eligible
HMO Institutional	-	HMO	HMO Institutional
Local PPO	-	Local PPO	-
Local PPO Chronic or Disabling Conditions	-	Local PPO	Local PPO Chronic or Disabling Conditions
Local PPO Dual-Eligible	-	Local PPO	Local PPO Dual-Eligible
Local PPO Institutional	-	Local PPO	Local PPO Institutional
MSA	MSA	-	-
PFFS	PFFS	-	-
Regional PPO	Regional PPO	-	-
Regional PPO Chronic or Disabling Conditions	-	-	Regional PPO Chronic or Disabling Conditions
Regional PPO Dual-Eligible	-	-	Regional PPO Dual-Eligible

Statistical Analyses

All regressions were performed in Stata version 16.1. For our primary analysis, regression specifications were of the following form:

© 2023 Schwartz AL et al. *JAMA Health Forum.*

- Difference-in-differences model:

$$y_{it} = \theta_i + \tau_t + \beta_1\, CUT_i \times WASHOUT_t + \beta_2\, CUT_i \times POST_t + \epsilon_{it} \quad (1)$$

where i indexes the county, t indexes the year, y_{it} is MA penetration θ_i is a vector of county fixed effects, τ_t is a vector of year fixed effects, CUT_i is a binary indicator for whether a county experienced a benchmark cut in the 75th percentile or above, $WASHOUT_t$ is an indicator for the ACA benchmark phase-in period (2012-2016) and $POST$ is an indicator for the post-phase in period (2017 or later), and ϵ_{it} is the error term. β_2 is the coefficient of interest corresponding to our effect estimate. In sensitivity analyses, we replaced CUT_i with alternate binary indicators for larger benchmark cuts, dichotomized at the median benchmark cut, or the 90th percentile benchmark cut. We also employed the ACA-induced benchmark cut, a continuous measure of benchmark cut exposure, in place of the binary CUT_i measure. The continuous measure ranged in value from 0 to 1, where 1 would represent a 100% cut. In another sensitivity analysis, we removed the β_1 term and redefined $POST_t$ period as 2012 onward. We used robust variance estimators to account for clustering within counties.

- Parallel trends testing and adjustment:

$$y_{it} = \theta_i + \tau_t + \gamma\, CUT_i \times year_t + \sum_{k=2012}^{2019} \mu_k * I(t = k \cap CUT_i = 1) + \epsilon_{it} \quad (2)$$

where $year_t$ is the continuous year indexed to 2011, γ is the difference in linear time trend between treatment and comparison groups prior to the ACA.[4] The mean of μ_k during the ACA period corresponds to the difference-in-differences estimate after adjustment for linear, non-parallel time trends in the pre-ACA period.

For our secondary analysis, regressions were of a similar form, but with payment rates as the outcome y_{it} and i indexing county-plan type.

eReferences

1. Monthly Contract and Enrollment Summary Report. CMS. https://www.cms.gov/Research-Statistics-Data-and-Systems/Statistics-Trends-and-Reports/MCRAdvPartDEnrolData/Monthly-Contract-and-Enrollment-Summary-Report. Published December 1, 2021. Accessed February 17, 2023.
2. Monthly Enrollment by Contract. CMS. https://www.cms.gov/Research-Statistics-Data-and-Systems/Statistics-Trends-and-Reports/MCRAdvPartDEnrolData/Monthly-Enrollment-by-Contract. Published December 1, 2021. Accessed February 17, 2023.
3. Special Needs Plan (SNP) Data. CMS. https://www.cms.gov/Research-Statistics-Data-and-Systems/Statistics-Trends-and-Reports/MCRAdvPartDEnrolData/Special-Needs-Plan-SNP-Data. Published December 1, 2021. Accessed February 17, 2023.
4. Bilinski A, Hatfield LA. Nothing to see here? non-inferiority approaches to parallel trends and other model assumptions. arXiv. https://arxiv.org/abs/1805.03273. Updated January 17, 2020. Accessed November 1, 2021.

© 2023 Schwartz AL et al. *JAMA Health Forum.*

eTable 1. Enrollment Sensitivity Analysis

Panel A: sensitivity analyses, weighted by Medicare population

	(1) Primary analysis	(2) Sensitivity analysis: exposure is benchmark cut > median	(3) Sensitivity analysis: exposure is benchmark cut > 90th percentile	(4) Sensitivity analysis: linear adjustment for non-parallel time trends	(5) Sensitivity analysis: continuous exposure
Effect estimate, binary exposure	0.017	0.341	0.231	0.264	
	(-1.175 – 1.210)	(-0.607 – 1.290)	(-1.596 – 2.057)	(-1.162 – 1.689)	
Effect estimate, continuous exposure					0.041
					(-0.047 – 0.129)
Differential linear trend, pre-ACA				-0.029	
				(-0.214 – 0.156)	
Adjusted R-squared	0.675	0.675	0.675	0.675	0.676

*** p<0.01, ** p<0.05, * p<0.1. 95% Confidence Interval in parentheses.

© 2023 Schwartz AL et al. *JAMA Health Forum.*

Panel B: sensitivity analyses, unweighted

	(1) Sensitivity analysis: primary analysis without weighting	(2) Sensitivity analysis: exposure is benchmark cut > median	(3) Sensitivity analysis: exposure is benchmark cut > 90th percentile	(4) Sensitivity analysis: linear adjustment for non-parallel time trends	(5) Sensitivity analysis: continuous exposure
Effect estimate, binary exposure	2.691*** (2.050 – 3.333)	1.591*** (1.009 – 2.172)	2.295*** (1.364 – 3.226)	1.588*** (0.705 – 2.472)	
Effect estimate, continuous exposure					0.174*** (0.132 – 0.217)
Differential linear trend, pre-ACA				0.130*** (0.019 – 0.240)	
Adjusted R-squared	0.526	0.523	0.522	0.527	0.526

*** p<0.01, ** p<0.05, * p<0.1. 95% Confidence Interval in parentheses.

© 2023 Schwartz AL et al. *JAMA Health Forum.*

Panel C: sensitivity analyses, weighted by Medicare population, no washout period

	(1) Sensitivity analysis: primary analysis without washout period	(2) Sensitivity analysis: exposure is benchmark cut > median	(3) Sensitivity analysis: exposure is benchmark cut > 90th percentile	(4) Sensitivity analysis: linear adjustment for non-parallel time trends	(5) Sensitivity analysis: continuous exposure
Effect estimate, binary exposure	0.113<br>(-0.731 – 0.958)	0.225<br>(-0.449 – 0.899)	0.098<br>(-1.215 – 1.411)	0.287<br>(-0.615 – 1.189)	
Effect estimate, continuous exposure					0.031<br>(-0.033 – 0.094)
Differential linear trend, pre-ACA				-0.029<br>(-0.214 – 0.156)	
Adjusted R-squared	0.675	0.675	0.675	0.675	0.676

*** p<0.01, ** p<0.05, * p<0.1. 95% Confidence Interval in parentheses.

© 2023 Schwartz AL et al. *JAMA Health Forum.*

eTable 2. Payment Sensitivity Analysis

Panel A: sensitivity analyses, weighted by Medicare Advantage enrollment

	(1) Primary analysis	(2) Sensitivity analysis: exposure is benchmark cut > median	(3) Sensitivity analysis: exposure is benchmark cut > 90th percentile	(4) Sensitivity analysis: linear adjustment for non-parallel time trends	(5) Sensitivity analysis: continuous exposure
Effect estimate, binary exposure	-78.348***	-57.102***	-132.732***	-98.041***	
	(-94.482 – -62.213)	(-71.220 – -42.984)	(-159.079 – -106.385)	(-135.640 – -60.441)	
Effect estimate, continuous exposure					-8.756***
					(-10.462 – -7.049)
Differential linear trend, pre-ACA				2.292	
				(-1.945 – 6.529)	
Adjusted R-squared	0.358	0.332	0.395	0.366	0.410

*** p<0.01, ** p<0.05, * p<0.1. 95% Confidence Interval in parentheses.

© 2023 Schwartz AL et al. *JAMA Health Forum.*

Panel B: sensitivity analyses, unweighted

	(1) Sensitivity analysis: primary analysis without weighting	(2) Sensitivity analysis: exposure is benchmark cut > median	(3) Sensitivity analysis: exposure is benchmark cut > 90th percentile	(4) Sensitivity analysis: linear adjustment for non-parallel time trends	(5) Sensitivity analysis: continuous exposure
Effect estimate, binary exposure	-90.410***	-51.242***	-154.928***	-149.192***	
	(-97.204 – -83.616)	(-57.026 – -45.457)	(-165.096 – -144.759)	(-169.968 – -128.416)	
Effect estimate, continuous exposure					-7.932***
					(-8.380 – -7.484)
Differential linear trend, pre-ACA				6.797***	
				(4.349 – 9.245)	
Adjusted R-squared	0.362	0.352	0.368	0.366	0.371

*** p<0.01, ** p<0.05, * p<0.1. 95% Confidence Interval in parentheses.

© 2023 Schwartz AL et al. *JAMA Health Forum.*

eFigure. County MA Growth Versus Benchmark Cuts

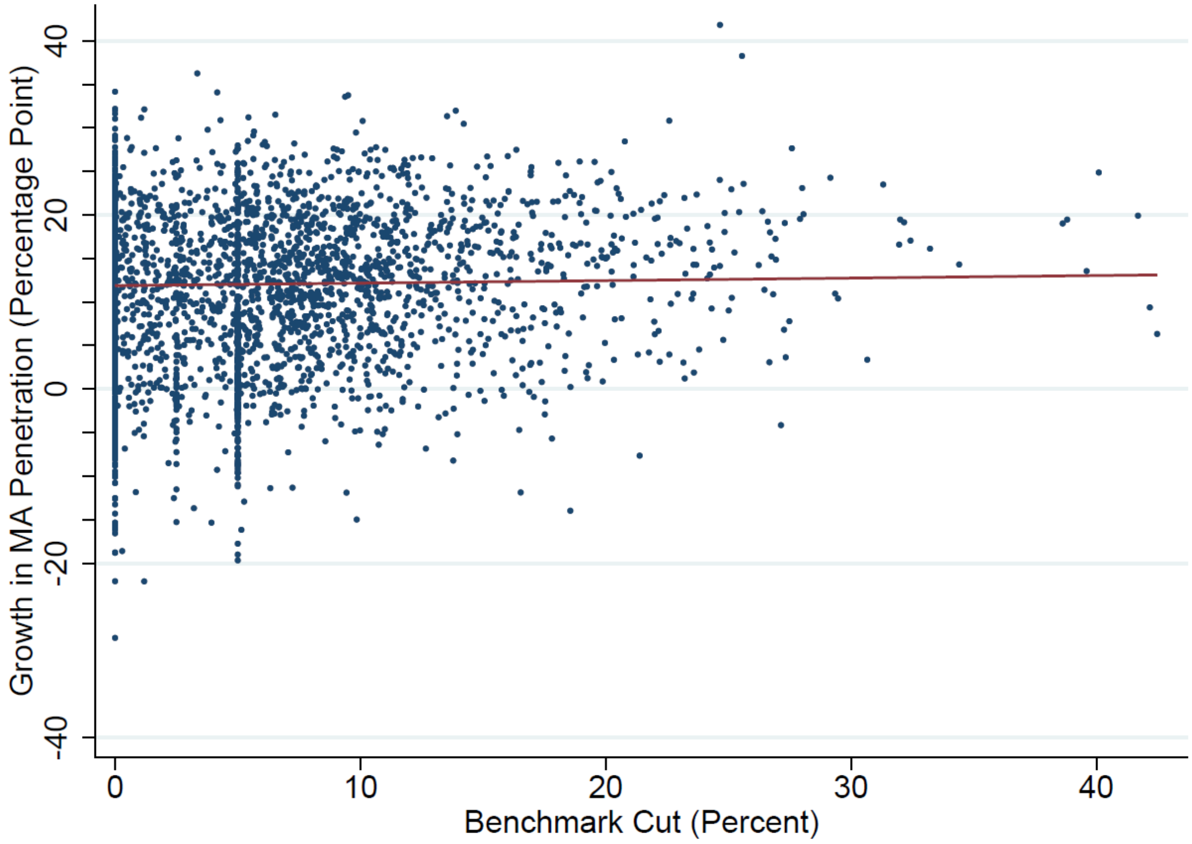

Note: Each dot represents one US county. Growth in MA penetration is the difference in MA penetration rates between 2011 to 2019. Benchmark cut is the ACA-induced benchmark cut, defined in eMethods. The red line represents the line of best fit.

© 2023 Schwartz AL et al. *JAMA Health Forum.*
