# Supplementary material for: Growth of Medicare Advantage After Plan Payment Reductions
Source: JAMA Health Forum. 2023 Jun 24;4(6):e231744. doi: 10.1001/jamahealthforum.2023.1744 (PMC10290750; doi:10.1001/jamahealthforum.2023.1744)
Supplement: Supplement 2. — Data Sharing Statement [file jamahealthforum-e231744-s002.pdf]

Data Sharing Statement

Schwartz. Growth of Medicare Advantage After Plan Payment Reductions. *JAMA Health Forum*. Published June 24, 2023. doi:10.1001/jamahealthforum.2023.1744

Data
Data available: Yes
Data types: Data (not involving human participants)
How to access data: Harvard Dataverse
When available: With publication

Supporting Documents
Document types: Statistical/analytic code
How to access documents: Harvard Dataverse
When available: With publication

Additional Information
Who can access the data: anyone
Types of analyses: for any purpose
Mechanisms of data availability: without investigator support
Any additional restrictions: none
